# Validation of the 5-D Itch Scale in Three Ethnic Groups and Exploring Optimal Cutoff Values Using the Itch Numerical Rating Scale

**DOI:** 10.1155/2021/7640314

**Published:** 2021-12-13

**Authors:** H. N. Cheung, Y. S. Chan, N. H. Hsiung

**Affiliations:** ^1^Department of Social Science, Hong Kong Metropolitan University, Hong Kong; ^2^Department of Applied Social Studies, Hong Kong Polytechnic University, Hong Kong; ^3^Department of Nursing, Asia University, Taiwan

## Abstract

**Background:**

Chronic pruritus is a common and distressing condition that has serious emotional and psychosocial consequences. Due to its subjective nature, self-report questionnaires are widely implemented as cost-effective measures to gauge the severity of chronic pruritus. The current study is aimed at validating the 5-D itch scale in three ethnic groups—Black, Asian, and Hispanic—with the well-validated Itch Numerical Rating Scale (NRS) and Worst Itch NRS (WI-NRS) and developing its cutoff value using receiver operating characteristics (ROC) and inspection of the area under the curve (AUC) across ethnic groups. At the same time, it is aimed at comparing the concurrent prevalence of itch and depression in these populations, who often form ethnic minorities in many countries. The current study addresses the knowledge gap of cultural adaptation of the 5-D pruritus scale for greater usage.

**Methods:**

Community samples of three ethnic groups were recruited from an online platform of Qualtrics and administered the self-report questionnaires of Itch-NRS, 5-D itch scale, and Patient Health Questionnaire-9 (PHQ-9) to measure their pruritus domains, itch intensity, depression screening, and its severity. Informed consent was obtained from all participants. Subgroup analysis was conducted, including concurrent validity and cutoff values compared between each ethnic group. Concurrent prevalence of itch and depression was evaluated using the cutoff value of Itch-NRS and PHQ-9.

**Result:**

A total of 2323 participants were included in the study. A significant positive correlation (*p* < 0.001) was found between the Itch-NRS, WI-NRS, and 5-D itch scale. The cutoff value of the 5-D itch scale was established for the three ethnic groups using ROC, with a cutoff value of Itch-NRS as a reference.

**Conclusions:**

The 5-D itch scale has demonstrated sound psychometric properties in three ethnic groups and is closely related to Itch-NRS. The analysis of the cutoff value of the 5-D itch scale suggests that different cutoff values should be considered to reduce the inflation of pruritus severity.

## 1. Introduction

Pruritus (or itch) has been an emerging but important topic in the area of dermatology that impacts the quality of life. It is identified as one of the most prevalent skin condition [1], signified by unpleasant sensations of burning and tingling. Itch is often followed by the urge to scratch as a reflex-like response [2], which leads to the itch-scratch cycle of reliving an intolerable itch [3] by scratching [4]. However, the itch-scratch cycle precedes the worsening of itch due to its serotonin-releasing nature for reward and further worsens lesion which sustains itch [5].

Approximately one-third of the population experiences itching weekly. When pruritus lasts over 6 weeks, it becomes chronic and thus is more difficult to treat [6]. Over 20 million Americans are reported to experience chronic pruritus [7]. A recent epidemiological study in Germany (*n* = 334) recorded over a third of patients experiencing pruritus, close to 90% of whom had chronic pruritus [8]. An equally high prevalence of 40% was documented in Japan [9] and a comparable 38% in a Polish study [10]. However, the prevalence has been scarcely reported in Asian populations. Many studies have focused on investigating it as a comorbid condition in outpatients with eczema and dermatitis [11] and pruritus in older populations [12]. Chronic pruritus imposes a large burden on patients' quality of life, comparable to that of chronic pain [13]. Patients with chronic pruritus have been found to have worsened health-related quality of life in both genders [14], particularly affecting daily activities, sleep patterns, and interpersonal relationships. It is also associated with depression [15] and other psychological disorders, such as anxiety and sleep disorders, due to the irritating feeling of itch and visible skin lesions. This results in a vicious cycle in which psychological stress may further exacerbate pruritus or pain [16] and the frequency of scratching. Psychiatric comorbidities such as parasitosis, hallucinations, and compulsive scratching syndromes and increased life stresses [17] are also prevalent in pruritus patients, and suicidal ideation is a commonly reported consequence. A large-scale cross-sectional study in 13 European countries (*N* = 3530) reported 14.1% depression prevalence among dermatological patients with pruritus [18].

Chronic pruritus relies on self-report assessments due to its subjective nature. The unidimensional Itch Numerical Rating Scale (Itch-NRS) has been developed as a patient-reported outcome to provide cost-efficient screening tools for measuring itch severity. Itch-NRS measures itch severity in the past 24 hours. It is also one of the most popular scales used in clinical settings and is commonly used as a reference to validate other pruritus scales. Itch-NRS has been validated in several languages with good psychometric properties (e.g., [19, 20]. It has also been further developed to become a one-item Worst Itch NRS (WI-NRS) that measures the peak itch severity in the past 24 hours in patients with atopic dermatitis [20]. Both had psychometric properties widely validated across populations, including reliability and validity [20, 21]. The scores of Itch-NRS range from no (0 points), mild (1–3 points), moderate (4–6 points), severe (7–8 points), to very severe (≥9 points) [22]. A clinical study on patients with chronic pruritus supported a score range of NRS to be 3-7-9, 4-6-9, and 4-7-9 with good correlation coefficients between NRS and VAS [23]. The cutoff value of Itch-NRS indicates a moderate level of symptoms is considered clinically symptomatic, while a cutoff value was yet to be developed for the WI-NRS. However, the NRS only provides an overall measure of severity while lacking a multidimensional evaluation of itch. To overcome this shortcoming, another popular self-report scale, the 5-D itch scale [24], a multidimensional measure of itch duration, degree, direction, disability, and distribution, has been validated extensively across clinical groups, such as patients with burns, psoriasis, and haemodialysis [22, 25, 26], with impressive concurrent validity. Despite its well-researched reliability and validity in various languages such as Spanish [27], Chinese [28], and Urdu [29], the cutoff value of the 5-D itch scale has not been fully explored among healthy community adults in various ethnic groups for screening purposes. Considering the high prevalence of pruritus on its own in general populations, a brief and valid scale that can also screen adults with and without pruritus could have potentially important clinical implications, especially in primary care settings. In addition, previous validation studies of the 5-D itch scale focused heavily on its validity and reliability. Hence, the cross-cultural comparison association between itch and depression in ethnic groups other than Caucasian, such as Asian, Black/African, and Hispanic, should be explored. The current study is aimed at validating the 5-D itch scale against Itch-NRS and WI-NRS on the concurrent validity and developing a cutoff value of the 5-D itch scale based on the reference of the Itch-NRS cutoff value. It is also aimed at providing further insight into the prevalence of itch and its possible cooccurring condition such as depression among the three ethnic groups using Itch-NRS and the Patient Health Questionnaire-9 (PHQ-9). The current study provides important exploratory results in the psychometric properties of the 5-D itch scale across ethnic groups as well as the prevalence of itch and other conditions across ethnic groups.

## 2. Materials and Methods

### 2.1. Participants and Procedures

A community sample of 2323 individuals across three ethnic groups worldwide, Asian (Chinese, Japanese, Korean, Indian, and other Asian ethnicity), Black, and Hispanic, was collected through an online platform by Qualtrics. Data collection lasted from 11 January to 12 February 2021. Potentially eligible participants above 18 who fit the three specified ethnic groups and could read English were contacted through email with an information sheet and consent form for their consideration. After providing written consent, participants were given a brief questionnaire to collect information on demographics, including age, gender, current skin diseases, and presence of chronic pruritus; a brief one-item questionnaire of the Itch-NRS; WI-NRS; and a 5-item 5-D itch scale. On the questionnaire, they were also assessed on their mental health state using depression scales as the PHQ-9.

### 2.2. Measures

#### 2.2.1. The Itch Numerical Rating Scale (NRS)

The Itch-NRS is a self-reported, brief, unidimensional itch intensity scale facilitating a Likert scale from 0 (no itch) to 10 (worst imaginable itch) for increasing self-perception of itch severity. With regard to pruritus, two separate scales are used. The Itch-NRS measures the itch on average within the past 24 hours, and the Worst Itch NRS (WI-NRS) scale measures the worst itch in the past 24 hours. It has been validated to have good psychometric properties, including a high significant concurrent validity with visual analogue scale (VAS) (*r* > 0.8; *p* < 0.01) and a high test-retest reliability with intraclass correlation coefficient of 0.8 [19]. In the current study, a Pearson correlation of 0.87 (*p* < 0.001) was found between Itch-NRS and WI-NRS.

#### 2.2.2. The 5-D Itch Scale

The assessment of prevalence was conducted using the 5-D itch scale developed by Elman et al. [24], a brief self-report questionnaire including five domains of pruritus: duration, degree, direction, disability, and direction. The Chinese version has been translated into traditional Chinese characters [22] with good psychometric properties: Cronbach's alpha of 0.734, strong and significant correlation with a visual analogue scale, and sensitivity to change over time. Cronbach's alpha for the current sample was 0.93.

#### 2.2.3. Patient Health Questionnaire

The PHQ-9 is a widely used depression screening tool in primary care settings [30]. The nine-item self-report scale is documented to have acceptable concurrent validity. In differentiating depressed from nondepressed individuals, a cutoff value of 10 yielded high sensitivity (0.88) and specificity (0.85) [31, 32].

### 2.3. Data Analysis

SPSS 16 (IBM, 2020) was used for subgroup analysis. Chi-square tests of independence were performed to compare the differences between ethnic groups in demographic variables such as gender, mental health history, skin conditions, presence of itch by Itch-NRS cutoff of 4, presence of depression by PHQ-9, and presence of itch above 6 weeks. The significance level was set at *p* < 0.05.

Regarding the psychometric properties of the 5-D itch scale, the internal consistency of the 5-D itch scale was indicated by Cronbach's alpha. The 5-D itch scale was also compared with the Itch-NRS and WI-NRS for concurrent validity by Spearman correlation. The cutoff value of the 5-D itch scale was established by receiver operating characteristics (ROC) curve across three ethnic groups by comparing with the cutoff value of Itch-NRS as reference. Cutoff value of 4 of the Itch-NRS was tested against the 5-D itch scale, as it indicates moderate severity of pruritus and beyond this value would be clinically regarded as symptomatic pruritus [22]. The cutoff value was chosen because it was supported in validation of Itch-NRS in patients with other skin disease such as haemodialysis [22]. In addition, although Reich et al. [23] proposed the best cutoff value of 3 in Itch-NRS, a cutoff value of 4 was also supported in their study. ROC curves are indicators of combined sensitivity and specify all possible cutoff points with the area under the curve (AUC) measuring diagnostic accuracy. An AUC of ≥0.9 is regarded as “excellent,” 0.8-0.9 considered “good,” and 0.7-0.8 is considered “fair” [33]. The AUCs of the Itch-NRS and the 5-D itch scale were compared in the study. As the Itch-NRS was the sole diagnostic instrument, an optimal cutoff value was selected for the best trade-off between sensitivity and specificity.

## 3. Results

A total of 2323 questionnaires were completed at the viable platform for data collection. The descriptive statistics of participants from the three ethnic groups are listed in Table 1. Participants reported a mean age of 35.17 (standard deviation (SD) = 14.26). The population included 46.58% Asian, 27.38% Black/African, and 26.04% Hispanic. The majority of participants were female (65.5%). The study population comprised 339 participants (14.59%) with known mental disorders during the test period, including depression (39.23%), anxiety (21.82%), bipolar disorder (7.67%), and stress-related disorders (20.05%).

The chi-square test of independence of the differences between ethnic groups in demographic variables showed that there was a significant difference of participants with history of mental disorders among ethnic groups. A lower percentage (11.8%) of Black participants showed mental disorders when compared to Asian participants (12.4%) and Hispanic participants (21.5%) (*χ*^2^ (2, *N* = 2323) = 31.31, *p* < 0.001). Regarding skin conditions, 16.9% of Asian participants perceived a presence of adverse skin condition. Among them, over half reported eczema (51.9%). A similar percentage of 13.2% and 14.9% of Black and Hispanic participants, respectively, reported the presence of skin condition, while eczema was also the most common condition, accounting for around a third prevalence (*χ*^2^ (18, *N* = 2323) = 48.06, *p* < 0.001).

To test the occurrence of pruritus and depression, the prevalence of itch and depression was indicated by the percentage of the sample who scored above the cutoff of Itch-NRS and PHQ-9, respectively, based on the cutoff values of NRS ≥ 4 and PHQ ≥ 10 [34]. A significant effect was found in the ethnic difference of itch by NRS ≥ 4 (*χ*^2^ (2, *N* = 2323) = 26.1, *p* < 0.001) and concurrent itch and depression. The Black group reported the highest percentage of 23.4%, followed by the Hispanic group (21.3%) and the Asian group (12.8%) (*χ*^2^ (3, *N* = 2323) = 37.53, *p* < 0.001) (Table 1).

### 3.1. Convergent Validity

Subgroup analysis of ethnic groups suggested a significant positive correlation (*p* < 0.001) from Spearman correlation between the one-item Itch-NRS, WI-NRS, and 5-D itch scale (Table 2). The result suggested good convergent validity and thus high agreement of both the Itch-NRS and WI-NRS with the 5-D itch scale regarding the measurement of general and peak pruritus using the 5-D itch scale.

### 3.2. Cutoff Value of the 5-D Itch Scale by ROC

**Figure 1:**
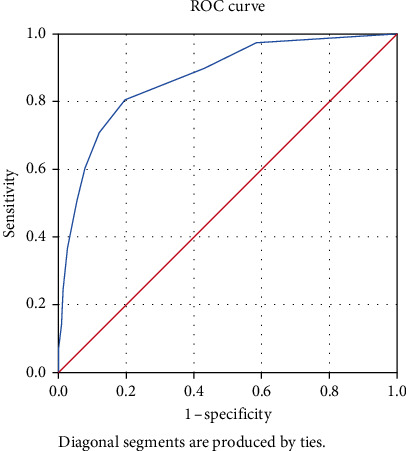
ROC curve and AUC for Asian sample.

On the basis of the NRS cutoff value of 4, the ROC curve for the Asian sample is shown in Figure 1. The AUC was 0.87 (95% CI: 0.84–0.90). A cutoff value of 8.5 yielded a sensitivity of 80.5% and 1-specificity of 80.3%.

**Figure 2:**
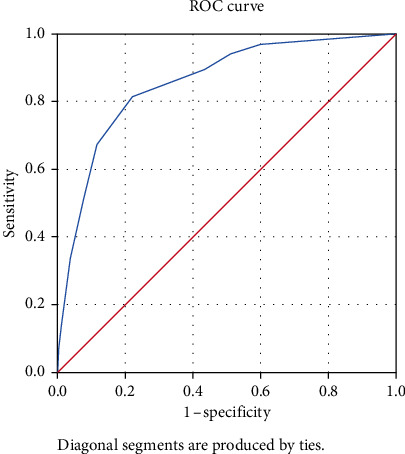
ROC curve and AUC for Black/African sample.

The ROC curve for the Black/African sample is shown in Figure 2. The AUC was 0.86 (95% CI: 0.83–0.89). A cutoff value of 8.5 yielded a sensitivity of 81.4% and 1-specificity of 77.8%.

**Figure 3:**
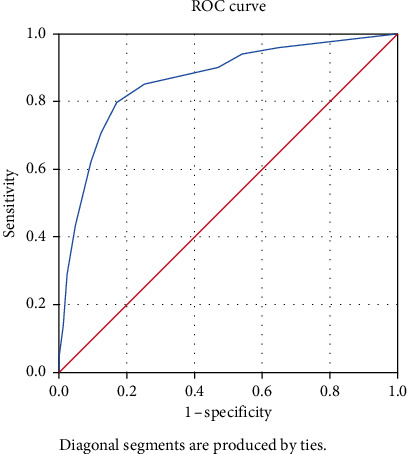
ROC curve and AUC for Hispanic sample.

The ROC curve for the Hispanic sample is shown in Figure 3. The AUC was 0.86 (95% CI: 0.84-0.90). A cutoff value of 9.5 yielded a sensitivity of 79.7% and 1-specificity of 83.0%.

## 4. Discussion

This study is aimed at exploring the psychometric properties of the 5-D itch scale and its cutoff value across community Asian, Black, and Hispanic samples using the cutoff value of Itch-NRS as a reference scale. It is also aimed at comparing the occurrence of itch and depression using the cutoff value of Itch-NRS and PHQ-9 across the three ethnic groups.

The demographic characteristics of the samples reported a significant variation in self-report of itch, depression, and adverse skin conditions. In coherent with the COVID situation of mental health, the prevalence of depression increased to a third of the sample in the Asian group and over half in the Hispanic group. The result is consistent with the finding that Hispanic adults experienced a greater level of psychosocial stress due to insufficient food and stable living conditions than adults in other ethnic groups [35]. Subsequently, the worsening in mental health could fuel the itch-psyche association as reflected in the cooccurring depression itch condition. It aligns with the positive association between chronic pruritus and depression [36]. Using the cutoff value of the Itch‐NRS ≥ 4 and PHQ‐9 ≥ 10, the Black/African group reported the highest percentage of itch and concurrent itch and depression, followed by the Hispanic group. This finding is coherent with that reported by Ruprecht et al. [37] that Black and Latin people are more vulnerable to mental distress, possibly due to being in a more disadvantaged economic position. At the same time, they are also at a higher risk of physical illnesses such as diabetes and unsuppressed HIV viral load, which could also contribute to self-reported pruritus [38]. The current study thus highlights the extra care in mental and itch areas required for ethnic minorities, especially for Black and Hispanic people across the world in dermatology and psychology.

The results of the current study reported good psychometric properties in a community sample of three ethnic groups, as indicated by the significantly high concurrent validity of the 5-D itch scale with the Itch-NRS and WI-NRS. The findings show that the multidimensional 5-D itch scale is a useful screening tool to reflect the severity of itch among the three ethnic populations. Using NRS ≥ 4 as a reference, the cutoff value of the 5-D itch scale identified the same cutoff value for the Asian and Black/African groups (8.5) but a higher cutoff value for the Hispanic group (9.5), yielding similar high sensitivity and specificity (~0.8). This highlights the importance of cultural adaptation of cutoff values of the 5-D itch scale to increase the accuracy of classifying individuals with and without itch. In particular, Hispanic ethnic groups may report a more elevated itch score and using the same cutoff value as for other ethnic groups would result in an inflation of itch identification. In addition, the cutoff values of the 5-D itch scale across ethnic groups in the current study are lower than the score of reported moderate pruritus (i.e., 12) in previous studies [22]. A more conservative cutoff value might be an advantage in community screening to lower the chance of false negative.

The current study addresses the important gap in the literature in cross-cultural validation of the 5-D itch scale, as our results indicate that a cutoff value is usually not established or validated across ethnic groups. It also opens up the opportunity for further investigation into the cutoff scores across cultures in comparison to the clinical population. It has important implication in developing a valid screening tool for pruritus in community sample potentially in primary care settings.

There are some potential limitations of this study. Recruiting samples from a crowdsourcing Qualtrics platform has been reported to be skewed towards a more elevated score of mental health than the general population. In the current study, the demographics of the sample showed slight skewness towards males. A clinical sample is needed as a comparison group to establish the cutoff value of the 5-D itch scale in the future. Finally, an elevated score of the PHQ-9 resulting from COVID-19 might influence the result of the concurrent prevalence of individuals with depression and itch.

## 5. Conclusion

In the validation studies, the 5-D itch scale was valid and reliable among different ethnic groups. A cutoff score with high sensitivity and specificity has been established in the 5-D itch scale using the Itch-NRS as a reference. The study is also coherent with previous literature that Black and Hispanic people are more vulnerable to concurrent itch and depression than Asians.

## Figures and Tables

**Table 1:** Descriptive statistics of three ethnic groups.

Variables	Asian (*n* = 1082)	Black/African (*n* = 636)	Hispanic (*n* = 605)	*χ* ^2^
Age Mean (SD)	37.0 (14.74)	34.0 (14.5)	33.1 (12.7)	—
Gender *n* (%)				
Female	700 (64.7)	422 (66.4)	400 (66.1)	0.62
Male	382 (35.3)	214 (33.6)	205 (33.9)	
History of mental disorders *n* (%)				
Yes	134 (12.4)	75 (11.8)	130 (21.5)	31.31^∗∗^
No	948 (87.6)	561 (88.2)	475 (78.5)	
Presence of skin diseases *n* (%)				
Yes	183 (16.9)	84 (13.2)	90 (14.9)	4.31
No	899 (51.3)	552 (86.8)	515 (85.1)	
Current skin diseases *n* (%)				
Acne	14 (7.6)	3 (3.6)	10 (11.1)	48.06^∗∗^
Atopic dermatitis	95 (51.9)	24 (28.6)	30 (33.3)	
Dryness	17 (9.3)	6 (7.1)	11 (12.2)	
Psoriasis	4 (2.2)	2 (2.4)	5 (5.6)	
HS	1 (0.5)	1 (1.2)	1 (1.1)	
Hives	3 (1.6)	1 (1.2)	0 (0.0)	
Rashes and fungus infection	12 (6.6)	2 (2.4)	2 (2.2)	
Lupus	0 (0.0)	2 (2.4)	0 (0.0)	
Others and unspecified	37 (20.2)	43 (51.2)	31 (34.4)	
Presence of depression PHQ‐9 ≥ 10 *n* (%)				
Yes	334 (30.9)	287 (45.1)	309 (51.1)	75.45^∗∗^
No	748 (69.1)	349 (54.9)	296 (48.9)	
Presence of pruritus Itch‐NRS ≥ 4 *n* (%)				
Yes	261 (24.1)	226 (35.5)	182 (30.1)	26.10^∗∗^
No	821 (75.9)	410 (64.5)	423 (69.9)	
Presence of both depression and pruritus *n* (%)				
Yes	138 (12.8)	149 (23.4)	129 (21.3)	37.53^∗∗^
No	944 (87.2)	487 (76.6)	476 (78.7)	
Presence of itch symptoms over 6 weeks *n* (%)				
Yes	196 (18.1)	107 (16.8)	94 (15.5)	1.86
No	886 (81.9)	529 (83.2)	511 (84.5)	

^∗∗^ *p* < 0.001.

**Table 2:** Spearman correlation between Itch-NRS and WI-NRS with 5-D itch scale.

	Itch-NRS/5-D	WI-NRS/5-D
Asian	0.67^∗^	0.69^∗^
Black/African	0.64^∗^	0.63^∗^
Hispanic	0.67^∗^	0.68^∗^

*p* < 0.001.

## Data Availability

The data is available upon request. Please contact the corresponding author at cheunghn@hkmu.edu.hk.
