# Corrigendum to “Validation of the 5-D Itch Scale in Three Ethnic Groups and Exploring Optimal Cutoff Values Using the Itch Numerical Rating Scale”

**DOI:** 10.1155/2022/9759214

**Published:** 2022-08-13

**Authors:** H. N. Cheung, Y. S. Chan, N. H. Hsiung

**Affiliations:** ^1^Department of Social Science, Hong Kong Metropolitan University, Hong Kong; ^2^Department of Applied Social Studies, Hong Kong Polytechnic University, Hong Kong; ^3^Department of Nursing, Asia University, Taiwan

In the article titled “Validation of the 5-D Itch Scale in Three Ethnic Groups and Exploring Optimal Cutoff Values Using the Itch Numerical Rating Scale” [1], the authors requested the following correction to the acknowledgement statement:

Acknowledgments

The work described in this paper was substantially supported by the Katie Shu Pui Charitable Trust (KS 2020/03) and the Public and Social Policy Research Centre, which had been established and supported by a grant from the Research Grants Council of the Hong Kong Special Administrative Region, China (UGC/IDS16/18).
